# Investigating the Potential of By-Products from Clitoria and Borage Flower Infusions for Valorization: A Comparative Study

**DOI:** 10.3390/molecules31081335

**Published:** 2026-04-18

**Authors:** Nesa Dibagar, Anna Michalska-Ciechanowska, Alicja Kucharska-Guzik

**Affiliations:** 1 Department of Fruit, Vegetable and Plant Nutraceutical Technology, Faculty of Biotechnology and Food Science, Wrocław University of Environmental and Life Sciences, Chełmońskiego 37 Str., 51-630 Wrocław, Poland; anna.michalska@upwr.edu.pl (A.M.-C.); alicja.kucharska@spark-lab.pl (A.K.-G.); 2 Laboratorium Analiz Chemicznych Spark-Lab Sp. z o.o., Aleja Zwycięstwa 96/98, 81-451 Gdynia, Poland

**Keywords:** herbs, infusion, side stream, valorization, polyphenolic compounds, antioxidant capacity

## Abstract

This study evaluates the potential of marc, a by-product of clitoria (*Clitoria ternatea* L.) and borage (*Borago officinalis* L.) infusions, as a preliminary step toward their subsequent conversion into functional food ingredients. After infusion, the marc was collected and processed by carrier-assisted crushing, aqueous maceration, and subsequent separation into extract and residue fractions. The impact of flower pretreatment by milling and marc matrix modification by inulin and maltodextrin was studied on the physical (dry matter (DM), water activity, color), chemical (total phenolic content (TPC), sum of individual phenolic compounds, and antioxidant capacity), and solubility of the microencapsulated fractions. Inulin-formulated powders derived from intact flowers’ marc were characterized by higher dry matter, decreased water activity, and improved chemical profiles. Under these conditions, clitoria by-products exhibited mean dry matter 94.17 ± 0.20%, water activity 0.301 ± 0.003, TPC 3.285 ± 0.052 mg GAE/g DM, sum of individual phenolic compounds 6.267 ± 0.103 mg/g DM, and ABTS-determined antioxidant capacity 0.100 ± 0.001 mmol Trolox/g DM. For borage by-products under identical conditions, dry matter content (−1.60%), water activity (−12.62%), TPC (−39.82%), sum of individual phenolic compounds (−67.55%), and antioxidant capacity (−65.00%) were lower compared with clitoria by-products. An efficient extraction and stabilization approach can open opportunities for upcycling post-extraction herbal residues into high-value food ingredients.

## 1. Introduction

Herbal plants constitute a vital component of food, pharmaceutical, and cosmetic applications, supported by a long history of cross-cultural utilization [1]. They are rich in bioactive constituents, particularly phenolic compounds, including phenolic acids, flavonoids, anthocyanins, and other secondary metabolites, which play a key role in determining the chemical functionality of plant materials [2,3,4,5]. Within the food industry, herbal plants are widely utilized as spices, flavoring agents, natural colorants, and functional ingredients in food and beverage formulations [6,7]. In addition, they are incorporated as sources of natural antioxidants and preservatives, contributing to improved oxidative stability and extended shelf life of products [5,8]. Herbal-derived compounds are increasingly incorporated into diverse food products, including beverages (e.g., infusions and herbal teas) [9,10], bakery and confectionery products [11], dairy alternatives [12], and novel plant-based formulations [13], where they enhance sensory properties, nutritional value, and overall product quality.

Beyond food applications, herbal preparations have been used to address diverse health conditions, supported by substantial in vitro evidence and some in vivo studies. They are employed to alleviate neuropsychiatric disorders such as stress and insomnia [14], gastrointestinal disturbances [15], Alzheimer’s disease [16], and civilization diseases, including diabetes, obesity, and cardiovascular disorders [10,17]. Nevertheless, the safe and rational application of herbal products necessitates comprehensive elucidation of their mechanisms of action, potential adverse effects, contraindications, and interactions with conventional pharmaceuticals or functional foods [18].

Herbal infusions crafted from edible medicinal plants are consumed as daily beverages [9,10] by approximately two-thirds of the world’s population [19]. The consumption of herbal tea offers a simple and effective approach for combining sensory appeal with the preventive and therapeutic potential of medicinal plants. Similar to standard teas, herbal counterparts are mainly consumed in the form of an infusion of dried herbs brewed in hot water, a practice widely adopted in commercial settings, including the food industry, hotels, restaurants, and households. The paste-like waste left after herbal infusion preparation or color extraction, often regarded as marc, contains numerous bioactive compounds [20]. Despite the inherent richness of marc, its disposal remains a concern, as it leads to the loss of valuable bioactive constituents, including phenolic compounds. This overlooked resource represents a missed opportunity for enhancing resource efficiency and promoting circular bio-economy principles within the framework of sustainable development goals (SDGs) [21].

Maceration is a conventional and widely used extraction method for medicinal herbs and their by-products, based on immersing plant material in a solvent to promote cell wall disruption and the diffusion of soluble phytochemicals [22]. Despite its simplicity, maceration is often limited by low extraction efficiency and long processing times (hours to days). To address these limitations, the carrier-assisted crushing of plant material prior to maceration serves as a physical matrix modification approach. This method utilizes mechanical forces to disrupt plant cell walls, enhance physical rupture of the plant tissue, and improve matrix-carrier interfacial contact. Consequently, the accessible surface area is markedly increased during maceration, promoting greater water solubility and extractability of target bioactive compounds. Mechanical pretreatment prior to plant extraction has been successfully applied to obtain natural products, such as antioxidant phenolic compounds from bamboo leaves [23] and bay laurel leaves [24]. 

As examples of tea waste valorization, black tea waste fibers have been repurposed to produce instant tea, showcasing an innovative approach to resource utilization. The prepared instant tea retained significant chemical advantages, including high levels of antioxidant phenolic compounds, such as catechins, theaflavins, and thearubigins, which enhance health benefits. It also contains notable amounts of theanine and caffeine, contributing to its functional properties [20]. In another example, the quality of herbal dust extracts from green tea, hibiscus, and lemon balm obtained via ultrasound-assisted extraction was evaluated, focusing on the total phenolic content (TPC) and the phenolic profile of waste material. Optimized extraction yielded TPC and bioactive compounds, such as epigallocatechin gallate in green tea, neochlorogenic acid in hibiscus, and rosmarinic acid in lemon balm, comparable or superior to commercial extracts, with proven pharmaceutical activities [25]. This evidence confirms that the herbal dust extracts from green tea, hibiscus, and lemon balm are rich in bioactive compounds.

Among herbal plants, blue-flowering species, such as clitoria (*Clitoria ternatea* L.) and borage (*Borago officinalis* L.), have attracted considerable scientific interest owing to their unique phytochemical compositions and visually distinctive extracts. Clitoria, commonly known as butterfly pea, is an edible herb from the tropical and subtropical regions. Extracts from the blue petals of clitoria have a high content of polyphenols, mainly anthocyanins [10,26]. The U.S. Food and Drug Administration (FDA) has approved butterfly pea flower extract as a color additive for specific food applications [27]. The borage plant is native to the Mediterranean region and found in parts of Europe, North Africa, and South America. The flowers of the plant contain phenolic acids and flavonoid components [28,29]. Anthocyanins, flavonoid pigments responsible for the vivid blue colors of borage and clitoria flowers, contribute to their popularity as colorful herbal infusions [10,26,28,29].

This research addresses a critical gap. Although numerous studies have focused on extraction methods [29,30], identification of dominant bioactive compounds [31,32], and drying techniques [28,33] for clitoria and borage flowers, none have explored the potential of the marc remaining after their extraction. The main hypothesis is that these marcs still retain measurable bioactive compounds that can be recovered and microencapsulated as plant-based powders for the future development of novel food prototypes such as beverages. The infusion process was employed as a laboratory-scale analogue of industrial extraction operations. It served as a representative model for the generation and utilization of such plant-derived by-products. 

The originality of this investigation stems from its exploration of the valorization potential of clitoria and borage flower marc, generated following herbal infusion preparation. This study introduces a novel methodology involving carrier-assisted crushing of the marc prior to maceration, designed to support the secondary extraction process. Furthermore, a zero-waste approach is implemented, whereby the cellulosic residue remaining after marc extraction is repurposed for subsequent analyses, ensuring the complete utilization of the material without producing additional waste.

This study aimed to evaluate the potential of post-infusion marc derived from clitoria and borage flowers and to identify the more promising by-product for subsequent valorization. The assessment included physical properties (dry matter, color, and water activity), chemical composition (total phenolic content, sum of individual phenolic compounds, and antioxidant capacity), and instant properties (solubility).

## 2. Results and Discussion

### 2.1. Physical Property Assessments

#### 2.1.1. Dry Matter

**Figure 1:**
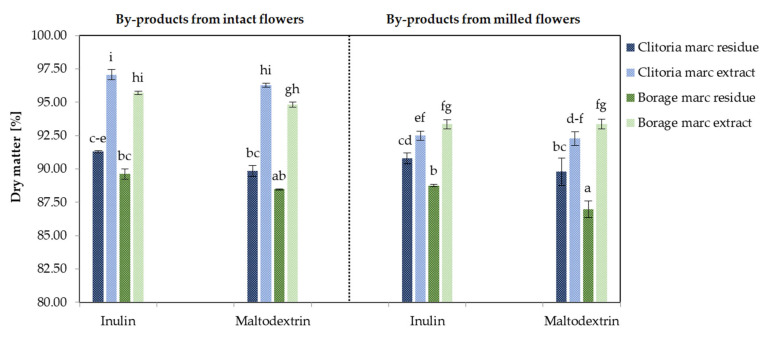
The dry matter [%] of clitoria and borage flower by-products; a, b, c, ...—different letters indicate statistically significant differences among samples (Tukey’s test, *p* < 0.05).

Figure 1 presents the dry matter content of by-product powders derived from clitoria and borage flowers, processed in various formats and with different carriers.

Focusing on the by-products of the clitoria flower, the dry matter content averaged 92.48% across all formats and carriers (range 89.78–97.06%). Averaging over fractions (marc extract and marc residue) and carrier type (inulin and maltodextrin), by-product powders from the intact flower showed higher dry matter content (mean 93.61%) than those from the milled format (mean 91.34%). This difference was statistically significant only for powders derived from the clitoria marc extract (*p* < 0.05). The higher dry matter content of powders from intact marc extracts is attributable to a higher content of non-volatile solids after the infusion. This higher solids feed may form a more cohesive matrix during freeze-drying, facilitating more complete moisture removal. By contrast, milling disrupts cells and promotes the leaching of soluble constituents during infusion preparation, leaving a lower solids extract that dries to a powder with a lower dry matter content.

Powders from clitoria marc extracts had significantly (*p* < 0.05) higher dry matter content than those from extraction residues (94.52% vs. 90.43%). This likely reflects compositional differences. Marc extracts are enriched in soluble non-volatile solids, whereas residues are dominated by insoluble cell wall polymers (cellulose-rich fiber and polysaccharides) [25,34]. These polymers bind water and create a fibrous network, impeding moisture diffusion and leaving higher residual water, thus resulting in a lower dry matter content in residue powders. Clitoria by-products processed with inulin showed a higher dry matter content than those processed with maltodextrin (92.91% vs. 92.04%), although the difference was not statistically significant.

Borage flower by-products exhibited a mean dry matter of 91.38% (range 86.98–95.70%), following the similar pattern of changes observed for clitoria by-products. Overall, by-product powders from the intact flower (mean 92.15%) showed higher dry matter than those from the milled flower (mean 90.61%). Within intact and milled borage formats, marc extract and marc residue powders differed significantly in dry matter content (*p* < 0.05) regardless of the carrier used in their formulation.

The higher dry matter content observed in samples processed with inulin may be attributed to its higher molecular weight and superior matrix-forming properties, which minimize structural collapse during freeze-drying and enhance moisture sublimation. Blackcurrant powders fortified with inulin during spray drying also had remarkably lower moisture content compared with maltodextrin [35].

Across all formulations, inulin-treated clitoria marc extract powder exhibited the highest dry matter content, significantly surpassing all other by-products (*p* < 0.05).

#### 2.1.2. Water Activity (*a_w_*)

**Figure 2:**
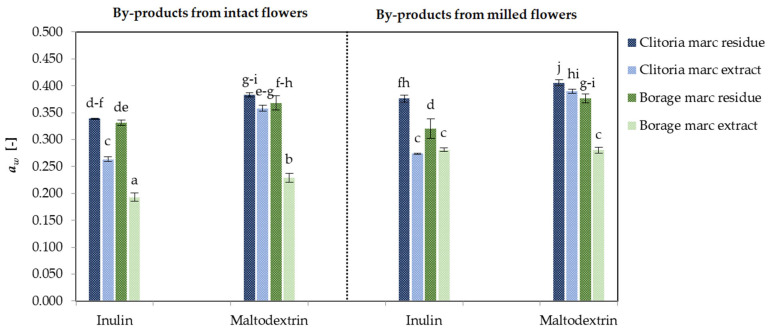
The water activity (*a_w_*) [-] of clitoria and borage flower by-products; a, b, c, ...—different letters indicate statistically significant differences among samples (Tukey’s test, *p* < 0.05).

Water activity is a fundamental parameter for characterizing food products, as it more accurately reflects the availability of free water for physical, chemical, and biological processes than moisture content alone [36]. The measurement of *a_w_* is critical for predicting food powder flowability, caking, and other handling properties during processing and storage, as these are highly dependent on the free water fraction in the matrix. Additionally, controlling water activity is essential for ensuring shelf life, since low *a_w_* values inhibit the growth of spoilage and pathogenic microorganisms in powders [37]. Figure 2 illustrates the *a_w_* of by-product powders derived from clitoria and borage flowers under different processing conditions. For clitoria by-products, *a_w_* values ranged between 0.264 and 0.406. Averaging over the by-product fractions (marc extract and marc residue) and carrier type (inulin and maltodextrin), powders from intact flower marc consistently exhibited lower *a_w_* than those from milled flowers (0.336 vs. 0.352). This trend corresponds to the higher moisture content recorded in milled-format powders (Section 2.1.1).

For clitoria by-products, the mean *a_w_* differed significantly between by-product fractions: 0.322 for marc extract powders versus 0.376 for marc residue powders (*p* < 0.05). Accordingly, marc extract powders exhibited lower *a_w_*. The increased *a_w_* in residue powders likely reflects their cellulose- and polysaccharide-rich matrix, which is more hygroscopic and contains higher moisture during drying and storage, yielding higher water activity [34].

The carrier type also affected *a_w_* in clitoria by-products, as the addition of inulin resulted in lower values of water activity than maltodextrin (0.313 vs. 0.384, *p* < 0.05). This reduction is likely associated with inulin’s β-configuration, which offers superior water-binding sites and restricts free water mobility more effectively than maltodextrin’s glucose chains [38,39].

For borage flower by-products, *a_w_* ranged from 0.193 to 0.377. The lowest *a_w_* (0.193) was observed in inulin-formulated powders derived from intact flower marc extracts, whereas the highest *a_w_* (0.377) occurred in maltodextrin-formulated powders from milled marc residues. This pattern mirrors that of clitoria. The microencapsulated marc extract derived from the intact flower and processed with inulin yielded the lowest *a_w_*, while marc residue powders obtained from the milled flower marc extraction with maltodextrin corresponded to the highest *a_w_*.

These results indicate that inulin is a suitable carrier for supporting the maceration of flower marc and enhancing powder stability for storage and use. Consistent benefits of formulation with inulin have been reported for freeze-dried blueberry juice [38] and pumpkin powder [40].

Across matrices, powders derived from clitoria marc averaged 1.17-fold higher *a_w_* than borage powders, potentially due to higher hygroscopicity. As reported by Handayani et al. [32], the freeze-dried clitoria flower extract exhibited hygroscopicity exceeding 10%, thereby excluding it from classification as a non-hygroscopic powder.

#### 2.1.3. Color

**Figure 3:**
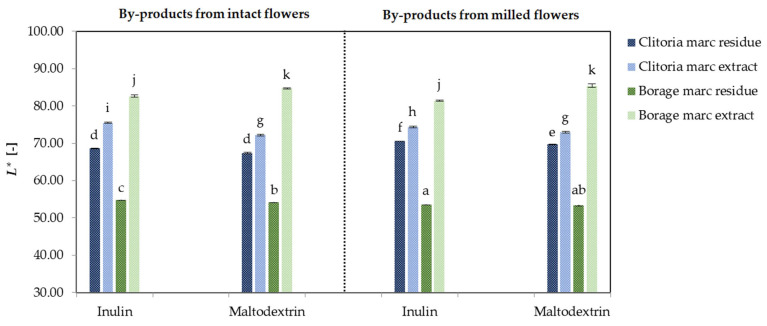
The L* [-] values of clitoria and borage flower by-products; a, b, c, ...—different letters indicate statistically significant differences among samples (Tukey’s test, *p* < 0.05).

Figure 3 demonstrates the L* values of by-product powders derived from clitoria and borage flowers, processed in various formats and with different carriers. Regardless of flower matrix and pre-infusion format, inulin increased the L* of the powders compared with maltodextrin (72.27 vs. 70.56, *p* < 0.05). This enhancement can be attributed to inulin’s formation of a white, amorphous glass structure, which increases diffuse backscattering and uniformly disperses pigments, reducing aggregation and apparent absorption, thereby boosting lightness [41]. Conversely, maltodextrin’s higher degree of polymerization may partially crystallize or aggregate during drying [42], entrapping moisture and yielding less uniform scattering, resulting in slightly lower L* values. In a study conducted by Michalska-Ciechanowska et al. [43], the cranberry juice powders containing inulin obtained after freeze-drying showed higher values of coordinate L* compared with maltodextrin-formulated powders.

In marc residues from both flowers, the fibrous structure characterized by higher bound water and internal shadowing, thus minimizes the carrier’s influence on L*. Similar results were observed when adding inulin to the tested oolong tea yogurts, which brightened the yoghurt color [44].

These findings align with the Principal component analysis (PCA)-derived groupings and the bar chart trends, reinforcing the interplay between carrier selection, matrix composition, and pigment characteristics in modulating powder lightness and color clustering.

**Figure 4:**
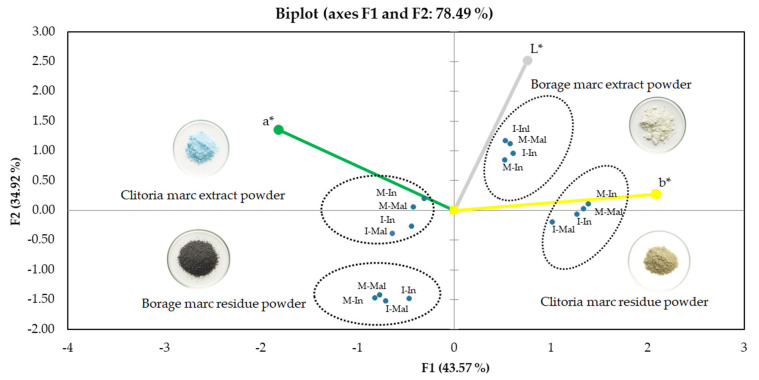
Biplot of PCA of colorimetric parameters of clitoria and borage flower by-products, I: intact, M: milled, In: inulin, Mal: maltodextrin.

The PCA biplot (Figure 4) explained 78.49% of the total variance, with the first two principal components contributing 43.57% (F1) and 34.92% (F2), respectively. The distribution of samples indicates that color differentiation is primarily governed by the b* (blue–yellow) coordinate along the positive F1 axis, while lightness (L*) is strongly associated with the positive F2 axis. The a* parameter contributes to the negative F1 direction, reflecting the red–green component as a secondary driver of variability.

Based on the dataset, borage marc extract powders are clearly positioned in the upper-right quadrant, corresponding to high L* values and slightly positive or near-zero b* values, indicating the lightest powders with low chromatic intensity. In contrast, borage marc residue powders are located in the lower-left quadrant, reflecting the lowest lightness values and moderate negative b* values, which together indicate dark bluish–greenish powders with reduced brightness.

Clitoria marc residue powders occupy the right-hand side but at lower F2 values, reflecting moderate lightness combined with distinctly positive b* values, which confirms their pronounced yellow coloration. Meanwhile, the clitoria marc extract powders are grouped on the left side of the biplot, consistent with their highest negative b* values and thus blue-dominated coloration. These separations underscore the influence of flower matrix composition (clitoria vs. borage) and processing fraction (marc extract vs. marc residue) on powder color characteristics.

All analyzed powders showed negative a* values in CIE Lab* color space, indicating a dominant greenish tonality, with the strongest expression observed in milled clitoria marc extracts. Along the b* axis, the clitoria marc extract and borage marc residue exhibited negative values, consistent with the blue color from anthocyanin pigments, whereas the clitoria marc residue displayed positive b* values, suggesting a shift to yellowish tones likely caused by anthocyanin degradation.

The characteristic blue color of clitoria flower by-products is primarily attributed to polyacylated anthocyanins known as ternatins [26]. In contrast, the different coloration observed in borage by-products is mainly associated with cyanidin-based anthocyanins. These compounds lack extensive acylation, which results in lower color stability typical of non-acylated anthocyanins [29]. The extent and intensity of color expression when using the developed powders in food product co-creation depends on the specific pH of the food matrix, as well as formulation and processing conditions.

### 2.2. Chemical Property Assessments

#### 2.2.1. TPC by the Folin–Ciocalteu Method

**Figure 5:**
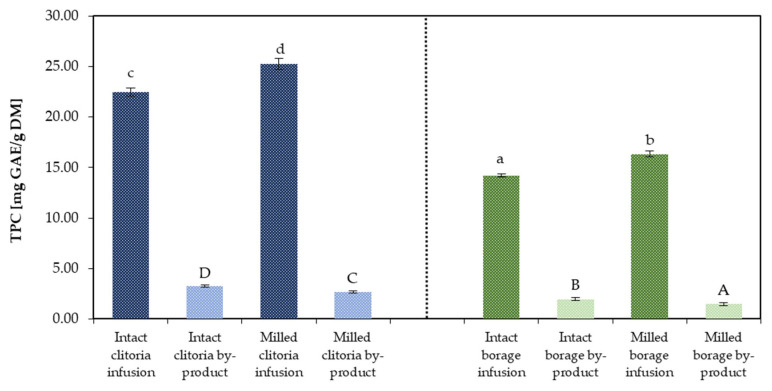
The total polyphenolic content (TPC) [mg GAE/g DM] determined by spectrophotometer in clitoria and borage flower infusions and their by-products; a, b, c, ...—different letters indicate statistically significant differences between samples (Tukey’s test, *p* < 0.05).

Figure 5 illustrates the TPC of clitoria and borage flower infusions and their corresponding by-products. The TPC of by-products was calculated by summing the TPC values of the marc extract and marc residue fractions (referred to as by-product) and subsequently averaging across formulations containing inulin and maltodextrin. For clitoria infusion, TPC reached 25.23 mg GAE/g DM and 22.46 mg GAE/g DM for milled and intact formats, respectively. These values are slightly lower than the TPC of 41.17 mg GAE/g DM reported under optimized extraction conditions in the literature [45]. The observed difference is likely attributable to variations in flower variety, extraction methodology, and processing conditions. The corresponding clitoria by-products exhibited TPC values of 3.25 mg GAE/g DM for the intact and 2.67 mg GAE/g DM for the milled flower, respectively. These values represented approximately 14% and 11% of the TPC of their respective infusions prepared from intact and milled flowers, respectively. This suggests that, although the majority of phenolic compounds are released into the infusion, residual phenolics remain in the marc and can still be recovered.

For borage, TPC of the milled- and intact-format flower infusions were 16.33 and 14.20 mg GAE/g DM, respectively, aligning with the ~16 mg GAE/g DM reported in raw borage leaves by Ceccanti et al. [46]. Across both flower matrices, milling significantly (*p* < 0.05) increased the TPC of infusions, consistent with a reduced particle size and greater surface area facilitating phenolic release.

The borage-derived by-products from intact and milled flowers contained TPC of 1.94 and 1.47 mg GAE/g DM, respectively. As observed for clitoria, these by-products represented approximately 14% and 9% of the TPC of their corresponding infusions prepared from intact and milled borage flowers, respectively. For both flower types, by-products obtained from intact matrices showed significantly higher TPC (*p* < 0.05) than milled matrices, likely because intact tissues restrict phenolic leaching during herbal infusion preparation, leading to greater phenolic content within the residual matrix.

**Figure 6:**
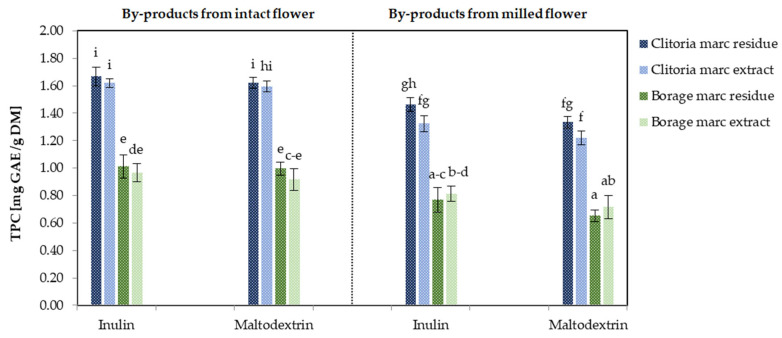
The total polyphenol content (TPC) [mg GAE/g DM] determined by Folin–Ciocalteu method in clitoria and borage flower by-products; a, b, c, ...—different letters indicate statistically significant differences among samples (Tukey’s test, *p* < 0.05).

Figure 6 illustrates the TPC values of by-product powders derived from clitoria and borage flowers, processed under various conditions. Focusing on clitoria by-product fractions, TPC values ranged from 1.22 to 1.67 mg GAE/g DM. Regardless of the carrier used, TPC was approximately 21% and 14% higher in powders derived from intact marc extracts and marc residues, respectively, compared with those obtained from milled-format flowers. This is likely because the reduced cellular disruption in intact flowers limits phenolic leaching during herbal infusion preparation, resulting in greater phenolic content in the marc residue.

When averaged across inulin- and maltodextrin-containing powders from both intact and milled formats, powders derived from clitoria marc extracts and marc residues did not differ significantly in TPC (1.52 mg GAE/g DM vs. 1.44 mg GAE/g DM, *p* > 0.05). These findings indicate that approximately half of the phenolic compounds present in the wet marc were recovered in the solvent (marc extract), while the remainder persisted in the solid marc residue. Implementation of a more intensive extraction strategy could enhance phenolic recovery into the solvent, thereby reducing residual phenolic content in the solid fraction following extraction. Regardless of the flower pre-infusion format (intact or milled), inulin-formulated marc extract and marc residue powders exhibited marginally, though not significantly, higher TPC than their maltodextrin-based counterparts. The TPC of milled clitoria by-product powders (marc extract plus marc residue) formulated with inulin was approximately 10% higher than that of the corresponding maltodextrin formulation, suggesting the improved encapsulation and protection of phenolics during maceration and drying. Michalska et al. [47] reported that spray-dried blackcurrant juice formulated with inulin contained a 21–48% higher total polyphenol content compared with powders produced with maltodextrin or inulin–maltodextrin blends. In addition, the anthocyanin content was approximately 27.6% greater in inulin-based formulations than in those containing maltodextrin under most microencapsulation conditions. 

In borage by-products, TPC values ranged from 0.65 to 1.01 mg GAE/g DM, with the highest levels observed in marc residues formulated with inulin (1.01 mg GAE/g DM) and the lowest in marc residues derived from milled borage flowers formulated with maltodextrin (0.65 mg GAE/g DM). On average across inulin- and maltodextrin-containing powders, by-products (marc extract plus marc residue) derived from intact flower matrices exhibited approximately 24% higher TPC than those obtained from milled borage. Carrier-related differences were also evident, with inulin-based formulations showing higher TPC than maltodextrin-based counterparts within the same intact marc residue configuration. Similar to the clitoria flower by-products, TPC of milled borage by-product powders (marc extract plus marc residue) formulated with inulin was approximately 11% higher than that of the corresponding maltodextrin formulation. This pattern mirrors that observed for clitoria by-products, indicating a consistent direction of change across flower matrices.

Notably, when averaged across milled and intact formats, the by-product derived from the clitoria flower exhibited approximately 1.7-fold higher TPC than that obtained from borage flowers.

#### 2.2.2. Sum of Individual Phenolic Compounds by HPLC Method

**Figure 7:**
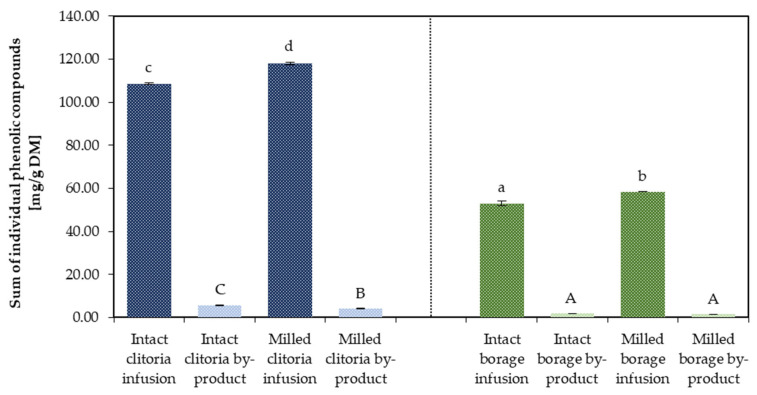
The sum of individual phenolic compounds [mg/g DM] determined by HPLC in clitoria and borage flower infusions and their by-products; a, b, c, ...—different letters indicate statistically significant differences among samples (Tukey’s test, *p* < 0.05).

The sum of individual phenolic compounds was assessed via high-performance liquid chromatography (HPLC) in clitoria and borage flower by-products, based on chromatographic separation profiles, with a representative chromatogram provided in the Supplementary Materials (Figures S1 and S2). Values for flower infusions and their corresponding by-products (marc extract plus marc residue) averaged across inulin- and maltodextrin-added powders are shown in Figure 7.

For clitoria, the sum of individual phenolic compounds was approximately 108.39 mg/g DM in infusions prepared from intact flowers compared with about 118.00 mg/g DM when milled flowers were used. This corresponds to an approximate 9% increase in phenolic release attributable to mechanical pretreatment by milling. A similar trend was observed for TPC measured by the Folin–Ciocalteu assay, with an increase of approximately 12% following milling.

In the case of borage, the sum of individual phenolics in the infusion increased from about 52.96 mg/g DM (intact flowers) to 58.39 mg/g DM (milled flowers), indicating roughly a 10% enhancement in phenolic release due to grinding. The increase in TPC determined by the Folin–Ciocalteu method was approximately 15%. Based on these comparisons, it can be inferred that preparing herbal infusions using intact flowers results in at least 9–10% of extractable phenolic compounds remaining in the spent marc under the studied conditions, relative to infusions prepared from ground plant material.

**Figure 8:**
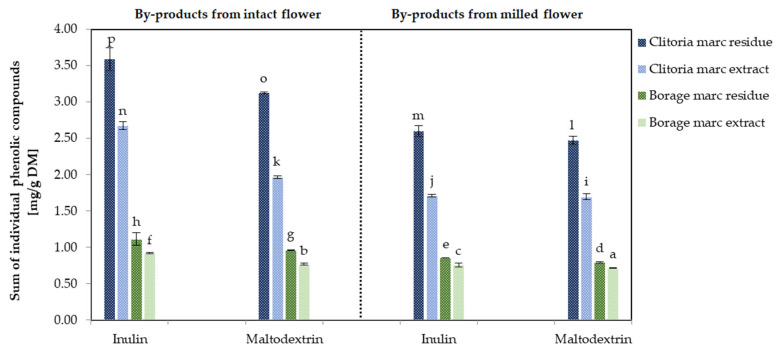
The sum of individual polyphenolic compounds [mg/g DM] determined by HPLC in clitoria and borage flower by-products; a, b, c, ...—different letters indicate statistically significant differences among samples (Tukey’s test, *p* < 0.05).

The HPLC results for by-product powders exhibited a consistent pattern of changes with the spectrophotometric data (Figure 8). Figure 8 shows the sum of individual phenolic compounds of by-product powders derived from flowers, processed in various formats and with different carriers. Specifically, clitoria by-products span 1.70–3.59 mg/g DM, whereas borage by-products fall within a narrower range of 0.716–1.11 mg/g DM (*p* < 0.05), reflecting inherent differences in the chemical composition and phenolic richness of the developed by-product powders from both flowers. The clitoria marc residue produced from intact flowers and formulated with inulin displayed the highest sum of individual phenolic compounds (3.59 mg/g DM). In agreement with the spectrophotometric data, inulin-formulated powders from the intact format contained higher sums of phenolics than maltodextrin-formulated powders and milled formats in both flowers. In the intact format, the sum of individual phenolics of the by-products (marc extract plus marc residue) formulated with inulin was 19% and 15% higher than that of the corresponding maltodextrin-containing powders for clitoria and borage flowers, respectively.

The sum of individual phenolic compounds quantified by HPLC in clitoria flower infusions and their by-product powders is expected, based on the extensive literature, to be predominantly influenced by polyacylated anthocyanins belonging to the ternatin family. Previous investigations report ternatins A-, B-, C-, and D-series (including ternatins A1–A3, B1–B4, C1–C5, and D1–D3) as the principal phenolic constituents of the flower extract. These compounds are delphinidin-based anthocyanins derived from delphinidin-3,3′,5′-triglucoside and acylated with *p*-coumaric acid and glucose moieties, conferring high molecular mass and enhanced stability [26,30,32,33]. Although full structural elucidation was not performed in the present study, the HPLC-quantified phenolic sum is therefore most plausibly driven by these ternatin pigments, with the literature frequently identifying ternatins B2, D1, and D2 as dominant contributors. In addition, minor contributions to the sum of individual phenolic compounds may arise from flavonoid glycosides, particularly quercetin-, kaempferol-, and myricetin-based derivatives, which are also commonly reported in clitoria flower matrices [28,29,31].

In contrast, the sum of individual phenolic compounds measured by HPLC in borage flower infusions and their corresponding by-product extracts likely reflects a more heterogeneous but less structurally complex phenolic profile, as documented in previous studies. The dominant contributors are expected to be phenolic acids, including rosmarinic, caffeic, chlorogenic, ferulic, and gallic acids, accompanied by flavonoids such as astragalin (kaempferol-3-O-glucoside), kaempferol-4-glucoside, rutoside (rutin), vitexin, myricetin derivatives, and the isoflavonoid daidzein. Anthocyanins present in borage are predominantly cyanidin-based, with cyanidin-3-glucoside and related derivatives commonly described as the major pigments, alongside minor amounts of other diglycosylated anthocyanins [29,31]. Consequently, the HPLC-derived phenolic sum for borage extracts is most reasonably attributed to the combined contributions of phenolic acids, flavonoids, and structurally simpler cyanidin-type anthocyanins rather than highly acylated anthocyanins such as those found in clitoria.

#### 2.2.3. Antioxidant Capacity (ABTS Assay)

**Figure 9:**
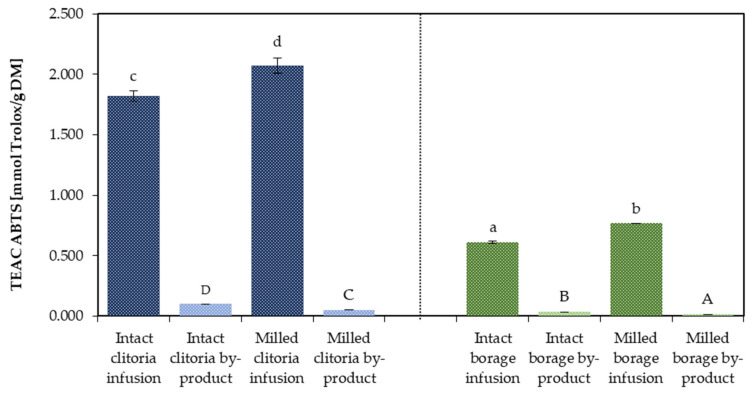
The antioxidant capacity [mmol Trolox/g DM] determined by the ABTS assay in clitoria and borage flower infusions and their by-products; a, b, c, ...—different letters indicate statistically significant differences among samples (Tukey’s test, *p* < 0.05).

Figure 9 provides a comparative evaluation of antioxidant capacity, as assessed by the ABTS assay, for infusions prepared from clitoria and borage flowers processed in milled and intact formats. The antioxidant capacities of their corresponding by-product (marc extract plus marc residue) averaged across formulations containing inulin and maltodextrin are also demonstrated in Figure 9. Clitoria flower infusions exhibited ABTS values of 2.07 mmol Trolox/g DM and 1.82 mmol Trolox/g DM for milled and intact formats, respectively, demonstrating a notable enhancement in antioxidant capacity in the infusion with milled flowers. The mean ABTS-determined antioxidant capacities of clitoria by-product powders were approximately 0.10 mmol Trolox/g DM for the intact and 0.05 mmol Trolox/g DM for the milled format, revealing an inverse trend where the intact format marc yielded a higher antioxidant capacity, consistent with observed patterns in TPC. These findings indicate that reducing particle size during extraction increases the release of antioxidant compounds into infusions, whereas intact format processing preserves more bioactive compounds in the resulting by-products.

For borage flower infusions, antioxidant capacity was recorded at 0.768 mmol Trolox/g DM for the milled format and 0.609 mmol Trolox/g DM for the intact format, again indicating a milling-induced increase. Similarly, borage by-products displayed mean antioxidant capacities of 0.029 mmol Trolox/g DM for the intact format and 0.015 mmol Trolox/g DM for the milled format, reinforcing the trend of elevated antioxidant capacity in intact format marc.

These findings highlight the significant (*p* < 0.05) impact of pre-infusion format on antioxidant profiles, with milling improving infusion potency and intact format marc exhibiting enhanced antioxidant capacity in by-products. Inter-flower comparisons of infusions indicate that the clitoria infusion possesses an antioxidant capacity approximately three-fold higher than that of the borage infusion, a difference potentially ascribable to the elevated phenolic compound content in clitoria flowers, as shown in Figure 6 and Figure 7.

The antioxidant capacity, determined by ABTS assay, in the by-products of clitoria and borage flowers were found to be comparable to those observed recently in the grape pomace of the *Grenache* variety (0.100 mmol Trolox/g DM) as a winemaking by-product [48] and the raspberry pulp of the *Rubus idaeus* L. variety (0.27 mmol Trolox/g DM) [49]. Moreover, the antioxidant capacity of nine medicinal plants from northern Chile with commercial potential was investigated by Trevizan et al. [50]. They reported ABTS values of 6–186 µmol Trolox/g DM (equivalent to 0.6–18.6 mmol Trolox/100 g DM). These findings underscore the high potential of borage and particularly clitoria for upcycling.

**Figure 10:**
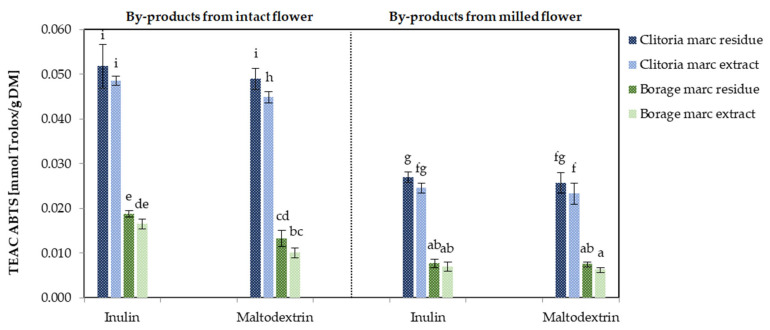
The antioxidant capacity [mmol Trolox/g DM] determined by the ABTS assay in clitoria and borage flower by-products; a, b, c, ...—different letters indicate statistically significant differences between samples (Tukey’s test, *p* < 0.05).

Figure 10 shows the ABTS antioxidant capacity of by-product powders derived from clitoria and borage flowers, processed in various formats and with different carriers. For clitoria by-product fractions, ABTS values ranged from 0.023 to 0.052 mmol Trolox/g DM. When averaged across inulin- and maltodextrin-containing formulations, marc residue powders exhibited significantly higher ABTS (*p* < 0.05) than the corresponding marc extract powders derived from clitoria in both milled and intact formats (0.050 mmol Trolox/g DM vs. 0.047 mmol Trolox/g DM for the intact format and 0.026 mmol Trolox/g vs. 0.024 mmol Trolox/g DM for the milled format). This observation suggests that a substantial proportion of antioxidant compounds remained associated with the fiber-rich marc residue, likely due to incomplete solvent penetration. Consequently, the application of more robust extraction techniques could improve antioxidant transfer into the solvent phase, thereby yielding a more enriched marc extract and a phenolic-depleted residue. Further, inulin-formulated clitoria by-products displayed, on average, about 6% higher ABTS than their maltodextrin counterparts, indicating a modest carrier-dependent enhancement in antioxidant preservation.

For borage by-products, ABTS values ranged from 0.006 mmol Trolox/g DM to 0.019 mmol Trolox/g DM. As observed for clitoria, the maximum (0.019 mmol Trolox/g DM) occurred for inulin-formulated powders from intact marc residue, whereas the minimum (0.006 mmol Trolox/g DM) was recorded for maltodextrin-formulated powders from milled marc extract. Thus, collecting marc from intact flowers and formulating with inulin produced a 2.4-fold higher ABTS capacity than the milled extract/maltodextrin counterpart. On average, clitoria by-products exhibited an ABTS capacity approximately 3-fold higher than borage by-products, underscoring matrix-dependent differences in antioxidant potential.

#### 2.2.4. Antioxidant Capacity (FRAP Assay)

**Figure 11:**
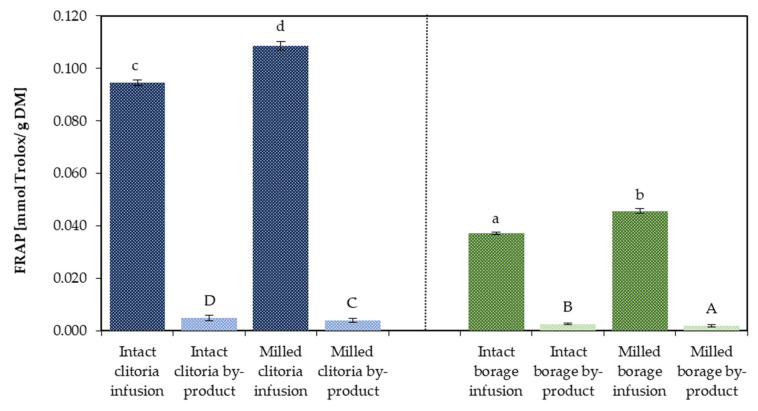
The antioxidant capacity [mmol Trolox/g DM] determined by FRAP assay in clitoria and borage flower infusions and their by-products; a, b, c, ...—different letters indicate statistically significant differences among samples (Tukey’s test, *p* < 0.05).

Figure 11 illustrates the ferric reducing antioxidant power (FRAP) of infusions and their corresponding by-products derived from clitoria and borage flowers in intact and milled formats. For clitoria infusions, FRAP values were 0.109 mmol Trolox/g DM for the milled format and 0.095 mmol Trolox/g DM for the intact format. The corresponding clitoria by-products contained, on average across inulin-and maltodextrin-added formulations, 0.005 (intact) and 0.004 mmol Trolox/g DM (milled). Borage flower infusion showed FRAP values of 0.046 and 0.037 mmol Trolox/g DM for milled and intact formats, respectively. The averaged FRAP-determined values in milled and intact format borage by-products were 0.002 and 0.003 mmol Trolox/g DM, respectively.

Within each flower, milling increased the antioxidant capacity of the infusions, whereas the intact formats yielded by-products with higher FRAP, showing an inverse trend. Cross-matrix comparison revealed that clitoria infusions possessed approximately 2.5-fold higher FRAP values than borage infusions, indicating stronger antioxidant potential in clitoria flower infusion under the tested conditions.

**Figure 12:**
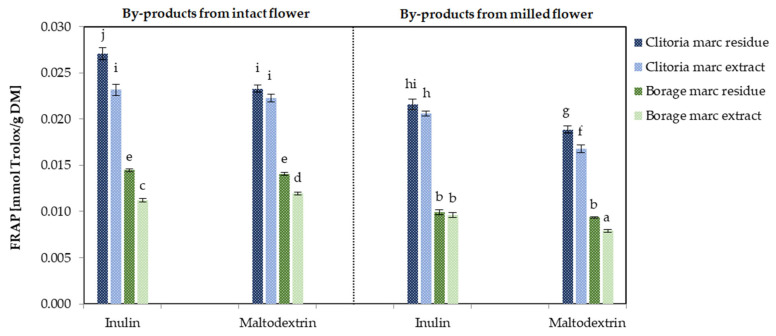
The antioxidant capacity [mmol Trolox/g DM] determined by FRAP assay in clitoria and borage flower by-products; a, b, c, ...—different letters indicate statistically significant differences among samples (Tukey’s test, *p* < 0.05).

Figure 12 shows the FRAP values of by-product powders derived from flowers, processed in various formats and with different carriers. The clitoria by-products exhibited FRAP values of 0.0039 and 0.0048 mmol Trolox/g DM for milled and intact flowers, respectively. The FRAP values for borage flower by-products were 0.0026 and 0.0018 mmol Trolox/g DM in milled and intact forms, respectively. These values are consistent with those reported for certain Indian medicinal plants, which exhibited FRAP values in the range of 0.0017–0.0052 mmol Trolox/g DM [51]. By-products derived from intact flowers exhibited higher FRAP-determined antioxidant capacity than those obtained from milled flowers across both flower matrices. Similar to the ABTS antioxidant capacity, marc residues from both clitoria and borage flowers showed significantly higher FRAP values than the corresponding marc extracts (*p* < 0.05). In clitoria, inulin-formulated by-products demonstrated a 9% and 15% increase in FRAP compared with maltodextrin-formulated by-products for the intact and milled formats, respectively. For intact borage flowers, inulin addition resulted in an 11% increase in FRAP values relative to maltodextrin. Consistent with the ABTS results, FRAP measurements further indicate that clitoria by-products exhibit approximately 2-fold higher antioxidant capacity than borage in intact flower formats.

### 2.3. Correlations of Antioxidant Assays and Phenolic Compounds

Pearson’s correlation was used to assess relationships between antioxidant assays (ABTS and FRAP) and phenolic measures (Folin–Ciocalteu and HPLC methods). The correlation matrix presented in Table 1 and Table 2 provides insights into the relationships among chemical properties of powders derived from the clitoria flower marc extract and residue fractions, respectively. In the marc extract powders, positive associations were observed between the sum of individual phenolic compounds (abbreviated as sum of phenolics) and TPC, FRAP, and ABTS, although these correlations did not reach statistical significance. In contrast, the marc residue powders exhibited stronger and statistically significant correlations between the sum of phenolics and TPC, FRAP, and ABTS. These results suggest that phenolic compounds are the primary contributors to antioxidant capacity in both processing fractions. Moreover, the significant correlation between the FRAP and ABTS assays implies that the antioxidant compounds in clitoria by-products not only scavenge ABTS free radicals effectively but also excel at reducing ferric ions. The strong correlation between the sum of individual phenolic compounds and TPC in both the marc extract and residue fractions indicates that the total phenolic content measurement reliably reflects the actual phenolic composition, thus validating TPC as a robust indicator of antioxidant potential in these samples.

Table 3 and Table 4 present the Pearson correlation matrices for powders derived from borage flower marc extract and marc residue, respectively. In the marc extract powders, the sum of individual phenolic compounds (abbreviated as sum of phenolics) showed positive but non-significant associations with spectrophotometric measures of antioxidant capacity. As expected, strong relationships were observed among the antioxidant assays, FRAP and ABTS, reflecting their common sensitivity to redox-active compounds.

In the borage marc residue fraction, the relationships were generally more pronounced. The sum of phenolics exhibited positive associations with TPC and the antioxidant capacity assays, indicating a more coherent distribution of phenolic compounds within this fraction. Notably, TPC showed a statistically significant association with FRAP, while its relationships with ABTS and the FRAP–ABTS pairing followed similar, albeit non-significant, trends. The highly significant correlations obtained in this study support the hypothesis that phenolic compounds contribute significantly to the total antioxidant capacity of herbal plants [51]. 

A strong correlation between TPC and antioxidant assays has been observed in 133 Indian medicinal plants [51] and across sixteen polyphenol-rich fruits and vegetables, respectively [52].

### 2.4. Instant Properties

#### Cold Water Solubility

**Figure 13:**
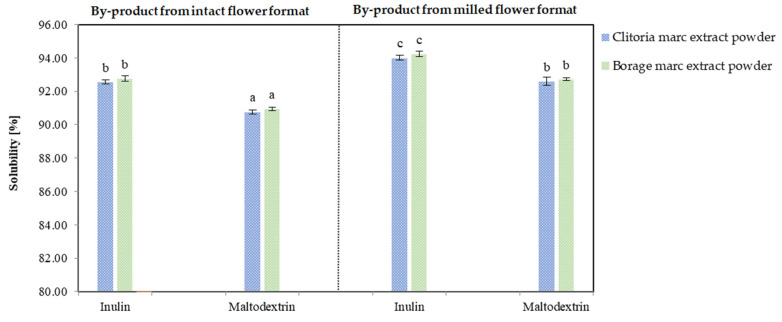
The solubility [%] in borage and clitoria marc extract powders; a, b, c—different letters indicate statistically significant differences between samples (Tukey’s test, *p* < 0.05).

The solubility analysis was conducted to assess the dispersibility of the plant-based powders in aqueous systems, as water solubility is a key indicator of their functional compatibility with a broad range of hydrophilic food matrices. A powder exhibiting satisfactory solubility in water is generally expected to display similar behavior in other liquid or semi-solid food systems, thereby facilitating its incorporation into diverse product formulations such as beverages, soups, or emulsions. Figure 13 reports the solubility of clitoria- and borage-derived marc extract powders. Across matrices and formats, all powders were highly soluble (greater than 90%), and the ordering was consistent regardless of flower matrix. In both flowers, milled-format marc extract powders were significantly more soluble than intact format powders (*p* < 0.05). For clitoria, the mean solubility was 93.30% for the milled format, whereas 91.66% for the intact format. With respect to carrier, inulin-formulated clitoria powders exhibited higher solubility than maltodextrin-formulated powders by roughly 2% (*p* < 0.05).

For borage, the highest solubility was observed for inulin-formulated powders from intact flowers (94.24%), whereas the lowest was recorded for maltodextrin-formulated powders from milled marc (92.75%). The identical ranking of treatments in clitoria and borage indicates that processing variables, flower format (milled vs. intact) and carrier type (inulin vs. maltodextrin), were the primary determinants of instant properties, while the botanical matrix exerts only a minor influence under these encapsulation conditions.

Mechanistically, milling increases cell disruption and extraction of low-molecular-weight, water-soluble constituents, while also reducing particle size and increasing porosity after freeze drying; together, these effects enhance wetting and dispersion, explaining the advantage of milled format over intact format. Regarding carriers, inulin formulations likely formed a more open, highly wettable amorphous network with greater capillary uptake than the denser glass typically produced by the maltodextrin grade used, yielding the reproducible inulin > maltodextrin difference within each format. Taken together, the data support the use of clitoria powders from milled marc extracts with inulin when rapid reconstitution is a priority, with only marginal trade-offs across species.

### 2.5. Limitations Related to Practical Food Applications

This study demonstrates the preliminary potential of post-infusion clitoria and borage by-products, including both marc extract and marc residue fractions, for prospective food applications. However, several limitations must be addressed in future studies before these materials can be developed into ready-to-use plant-based food ingredients.

Although the water activity of the by-product fractions fell within a range indicative of low free water availability, comprehensive microbiological evaluation is required to confirm the safety of the developed powders prior to their incorporation into food systems. Such assessment is essential to ensure compliance with food safety requirements.

The marc residue fractions from both flowers exhibited a substantial portion of the flower cell wall matrix (cellulose and fiber), inherently limiting water solubility and dispersion in aqueous media. Despite their phenolic compounds and antioxidant potential, the direct incorporation of marc residue powders into liquid or semi-liquid food products, such as beverages, functional drinks, or clear formulations, is challenging. Such applications require rapid solubilization or stable suspension to achieve acceptable sensory properties, visual clarity, and homogeneous distribution of bioactive compounds.

The application of more intensive extraction techniques, such as ultrasound-assisted extraction, can enhance the release of soluble bioactive compounds into the solvent, thereby increasing the techno-functional potential of the marc extract fraction while yielding a nutritionally depleted solid residue. Such a residue could alternatively be valorized as a cellulose-rich material for non-food applications, including composting or animal feed.

In addition to ensuring microbiological stability, chemical safety must be rigorously evaluated prior to any food applications of powders derived from borage flower by-products. In addition to FDA safety approval, other research further indicates that the aqueous extract from clitoria blue petals exhibits no cytotoxic effects on human fibroblast (IMR90) cells (LC_50_ > 900 μg/mL), provides protective effects on human erythrocytes, and inhibits oxidative damage to pBR322 plasmid DNA [26,27].

Since fresh borage flowers are known to contain pyrrolizidine alkaloids (PAs) [53], their unsupervised usage in the formulation of food products may raise toxicological concerns. Although the developed powders were produced from post-infusion borage marc, potentially resulting in lower PA levels than in fresh flowers, precise quantification through targeted analytical quantification, typically chromatographic methods such as LC-MS/MS, remains essential. Maximum permissible PA levels in food products are stipulated under EU Commission Regulation (EU) 2023/915 [54], offering regulatory benchmarks for exposure control in plant-derived materials. For borage by-product powders, a universally “safe” consumption threshold can be established only through direct PA quantification, as the risk hinges on the actual concentration in the final product.

## 3. Materials and Methods

### 3.1. Chemicals and Reagents

All solvents used were of analytical grade. Chlorogenic acid, gallic acid, and the Folin–Ciocalteu reagent were obtained from Sigma-Aldrich (Darmstadt, Germany). Sodium carbonate was purchased from Avantor Chemicals (Gliwice, Poland). ABTS (2,2′-azino-bis(3-ethylbenzothiazoline-6-sulfonic acid)) and potassium persulfate were supplied by Merck (Darmstadt, Germany). Hypergrade acetonitrile for HPLC-MS/MS analyses was sourced from Merck (Darmstadt, Germany), while HPLC-MS/MS-grade formic acid was obtained from VWR (Darmstadt, Germany). All other solvents and chemicals were purchased from Merck (Darmstadt, Germany). For formulation and microencapsulation purposes, maltodextrin (Pepees S.A., Łomża, Poland) and inulin (Beneo-Orafti, Oreye, Belgium) were used as carrier agents. Deionized water (Hydrolab HLP5, Straszyn, Poland; conductivity ≤ 4.3 µS/cm) was used for chromatographic analyses.

### 3.2. Material

The plant materials utilized in this study consisted of commercial dried flowers of borage (*Borago officinalis* L.) and clitoria (*Clitoria ternatea* L.) obtained from Dary Natury (Grodzisk, Poland).

### 3.3. Methods

#### 3.3.1. Preparation of Herbal Infusions and Marc Collection

**Figure 14:**
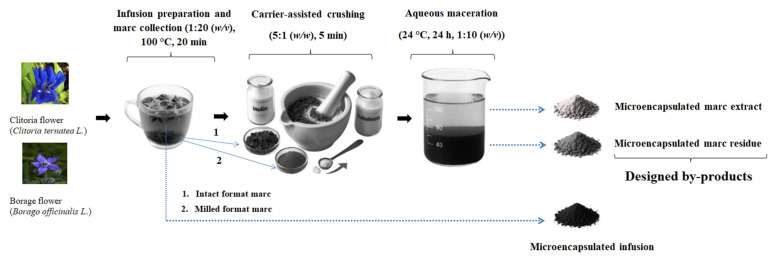
Schematic overview of the designation of clitoria and borage flowers’ post-infusion by-products.

Dried flowers of borage and clitoria underwent an aqueous extraction by simulating herbal tea brewing. For each flower type, 200 g of dried material was divided into intact and milled portions, with milling performed for 15 s using a grinder (TSM6A011W, Bosch, Munich, Germany). Both forms were extracted with 2 L of boiling water at 100 °C, stirred at 150 rpm for 20 min using a magnetic stirrer (IKA C-MAG HS 7, IKA-Werke GmbH & Co., KG, Staufen, Germany) (*n* = 2). Following the aqueous extraction, the marcs were separated from the infusions using Whatman No. 4 filter paper (Whatman, Maidstone, UK) and collected. The infusions from each flower format were subjected to centrifugation at 9000 rpm for 10 min at 4 °C (MPW 350R, MPW Med. Instruments, Warsaw, Poland) and filtration processes to serve as control samples for subsequent drying. Figure 14 presents a schematic overview of the infusion preparation and the designed by-products from dried clitoria and borage flowers. Within this study, the powdered marc extract and the marc residue are classified as the by-products.

#### 3.3.2. Designed By-Products

The marcs, collected right after the herbal infusion preparation, were subjected to a secondary extraction to recover their residual bioactive compounds. For each flower matrix, 50 g of the marc was first transferred to mortars in its wet format and underwent carrier-assisted crushing, incorporating 10 g of inulin or maltodextrin. Inulin and maltodextrin were chosen as food-grade carriers due to their safety, functionality, and proven performance in microencapsulation. Inulin, a GRAS fructan polysaccharide, is a functional food ingredient due to neutral sensory properties, favorable techno-functional behavior, and added prebiotic benefits [55]. Maltodextrin is also widely used for encapsulation because of its film-forming capacity, thermal stability, low cost, and ability to improve powder stability and handling without affecting sensory quality [56]. The mechanical disruption, conducted for 5 min, aimed to rupture cellular structures and enhance the extraction of entrapped bioactive constituents. This was followed by aqueous maceration, after which the crushed mixtures were transferred to containers with 600 mL of deionized water. The containers were sealed with Parafilm (Bemis Company, Inc., Neenah, WI, USA) to prevent contamination and incubated in a refrigerator at approximately 4 °C for 24 h, allowing for the diffusion and solubilization of residual bioactive compounds into the aqueous phase. The suspensions were then filtered through Whatman No. 1 paper filters (Whatman, Maidstone, UK) to separate the marc extract from the solid residue. The filtrates were collected, centrifuged at 9000 rpm for 10 min, and submitted to drying. Both marc extract and marc residue fractions of flowers were microencapsulated to obtain powders. This approach allowed subsequent analysis of their selected properties and facilitated assessment of the functionality between the solvent and the residual solid fraction. Consequently, the maceration residue was not discarded but instead microencapsulated. This comparison assessed whether residual phenolic compounds persisted in the marc residue. Microencapsulation of all liquid (infusions and marc extract) and solid (marc residue) fractions was conducted by freeze drying to (i) physically stabilize both liquid and solid fractions, (ii) improve handling, and (iii) protect bioactive compounds from oxidative, thermal, and environmental degradation during processing and storage [56].

#### 3.3.3. Microencapsulation by Freeze-Drying

The collected filtrates from flowers, marc, and the filtration solid residues were frozen for 24 h before freeze drying. Then, they were lyophilized using a laboratory freeze dryer (FreeZone, Labconco Corp., Kansas City, MO, USA; 24 h, 65 Pa, temperature of drying chamber and heating plate: −60 °C/25 °C) to obtain powdered products. The resulting freeze-dried powders were collected and vacuum-packed (MC 2006; Tepro SA, Koszalin, Poland) for subsequent physicochemical analyses. Based on the mass of powders obtained after freeze drying of marc extracts relative to the initial mass of crushed wet marc combined with the carrier, the extraction yield was calculated to range from 4 to 8% (*w*/*w*) for clitoria flower marc and from 3 to 6.7% (*w*/*w*) for borage flower marc.

#### 3.3.4. Physical Properties

##### Dry Matter (DM)

The dry matter of freeze-dried powders was determined using a vacuum oven method (Vacucell 111 Eco Line, MMM Medcenter Einrichtungen GmbH, Planegg/München, Germany) at 80 °C for 24 h (300 pa) [57] (*n* = 3). The results were reported as a percentage.

##### Water Activity (*a_w_*)

The water activity (*a_w_*) in powders was measured using the AQUA LAB DewPoint Water Activity Meter (Decagon Devices Inc., Pullman, WA, USA) at the temperature of 25 ± 2 °C (*n* = 3).

##### Color Coordinates (Lab*)

The color of by-product powders was quantitatively assessed using a Minolta colorimeter (CR-400, Osaka, Japan). The measurements were performed in the CIE Lab* color space, where L* represents lightness, a* corresponds to the red–green axis, and b* to the blue–yellow axis. The analyses were conducted under the standard illuminant D65 to ensure consistency and accuracy in color evaluation (*n* = 3).

#### 3.3.5. Chemical Properties

##### Extract Preparation

For chemical analysis (TPC, ABTS, and FRAP), 50 ± 0.2 mg of powder samples (calculated per DM) were dissolved in 1.5 mL of 80% acidified methanol with 1% HCL (*v*/*v*) (*n* = 2). Extracts were sonicated for 15 min at ambient temperature (25 °C) in an ultrasonic bath (Sonic 6D, Polsonic, Warsaw, Poland), followed by 20 min of continuous shaking on a rotator (Rotator Multi RS-60, Biosan, Riga, Latvia). They were left for 24 h at 4 °C and then re-sonicated (15 min). Next, the samples were centrifuged at 19,000 rpm for 15 min (MPW-251, MPW Med. Instruments, Warszawa, Poland), and the resulting supernatants were collected and used for the subsequent analyses.

##### Total Phenolic Content (TPC) by the Folin–Ciocalteu Method

Total phenolic content (TPC) was determined using the Folin–Ciocalteu method according to the procedure described by Horszwald and Andlauer [58] with some modifications. Briefly, 25 µL of appropriately diluted extract was transferred into a 96-well microplate. Subsequently, 250 µL of Folin–Ciocalteu reagent was automatically dispensed into each well using the microplate reader injector. After incubation for 10 min at room temperature, 25 µL of 20% (*w*/*v*) sodium carbonate (Na_2_CO_3_) solution was added to provide the alkaline conditions required for the reaction in the wells. The reaction mixtures were then incubated for an additional 20 min at room temperature to allow color development.

Absorbance was measured at λ = 750 nm using a Synergy H1 spectrophotometer (BioTek Instruments Inc., Winooski, VT, USA). Quantification was performed using a gallic acid calibration curve, and results were expressed as mg of gallic acid equivalent (GAE) per g of DM (mg GAE/g DM). All measurements were conducted in duplicate (*n* = 2).

##### Sum of Individual Phenolic Compounds by HPLC

The quantitative analysis of the sum of individual phenolic compounds was conducted using a high-performance liquid chromatography system (HPLC) equipped with a DAD detector (Shimadzu, Kyoto, Japan) and a Phenomenex Luna Omega column (Phenomenex, Torrance, CA, USA) (C_18_, 1.6 µm; 100 × 2.1 mm; 100 Å). The chromatographic separation was achieved via gradient elution with a mobile phase consisting of phase A (1% formic acid in water, *v*/*v*) and phase B (1% formic acid in acetonitrile, *v*/*v*) at a flow rate of 0.3 mL/min. The elution was performed using a step gradient as follows: 0–15 min, 95% A and 5% B; 15–55 min, 80% A and 20% B; 55–62 min, 60% A and 40% B; and 62.5–70 min, 95% A and 5% B. The injection volume was set to 5 µL, with the column temperature maintained at 30 °C and the autosampler temperature at 15 °C. Analytical wavelengths (190–700 nm) for the DAD detector were set according to the compound class being analyzed. The diluent was a 95:5 (*v*/*v*) mixture of phase A and phase B. Samples were prepared by taking 50 ± 2 mg of powders (calculated per DM) and making up the volume to 5 mL using diluent, sonicating for 3 min, centrifuging at 25 °C, and analyzing the filtrate. Standard solutions of chlorogenic acid were prepared for calibration, with working solutions diluted to 10 µg/mL for quantitative analysis, dissolved in the diluent. Quantification was performed using a chlorogenic acid calibration curve, and results were expressed as mg/g DM. All measurements were conducted in duplicate (*n* = 2). The calibration model exhibited excellent linearity, with a correlation coefficient (R) of 0.99997 and an adjusted coefficient of determination (adjusted R^2^) of 0.99994. Table S1 (Supplementary Material) presents the regression coefficients of the calibration model, including the slope and intercept, together with their standard errors, t-statistics, *p*-values, and 95% confidence intervals, used to evaluate the significance and reliability of the calibration curve. Furthermore, Table S2 (Supplementary Material) summarizes the analysis of variance (ANOVA) for the linear regression model of the chlorogenic acid calibration curve, including the degrees of freedom (df), sum of squares (SS), mean squares (MS), F-value, and overall model significance (*p*-value).

##### Antioxidant Capacity In Vitro by TEAC ABTS and FRAP Assays

The in vitro antioxidant capacity of clitoria and borage flower infusion and by-product powders was evaluated using the TEAC ABTS and ferric reducing antioxidant power (FRAP) assays.

For the TEAC ABTS assay, the ABTS radical cation (ABTS^•+^) solution was prepared by reacting ABTS with potassium persulfate (K_2_S_2_O_8_) and allowing the mixture to stand in the dark until complete radical stabilization. Prior to analysis, the ABTS^•+^ solution was diluted to obtain an absorbance of approximately 0.70 ± 0.02 at 734 nm. Aliquots of 10 µL of appropriately diluted samples were dispensed into 96-well microplates, followed by the automated addition of 290 µL of the ABTS working solution using the instrument injector. The reaction was carried out at 24 °C in the dark, and absorbance was measured at 734 nm after 6 min [59]. All measurements were performed in duplicate (*n* = 2).

The FRAP assay was performed according to the method described by Benzie and Strain [60]. Firstly, FRAP reagent was prepared freshly before analysis. Briefly, 10 µL of each extract was transferred into the wells. Subsequently, 10 µL of distilled water was added, followed by the addition of 200 µL of prepared FRAP reagent. The reaction mixture was left to stand for 10 min at room temperature, after which the absorbance was measured at 593 nm. All measurements were performed in duplicate (*n* = 2). A Synergy H1 spectrophotometer (BioTek Instruments Inc., Winooski, VT, USA) was employed to measure the absorbance. For both ABTS and FRAP assays, antioxidant capacity was quantified using external calibration curves constructed with Trolox. Standard Trolox solutions prepared by serial dilution (0.1 mM–10 mM) were analyzed under identical experimental conditions to those used for the ABTS and FRAP assays, and their linear regression equations were used to calculate antioxidant capacity. Antioxidant capacity values were calculated by interpolating absorbance measurements against the corresponding Trolox calibration curves and were expressed as Trolox equivalents. The results were expressed in mmol Trolox/g DM.

#### 3.3.6. Instant Properties

##### Cold Water Solubility

The cold water solubility of powders was performed in duplicate (*n* = 2) according to [61]. To 1 g of powder (calculated per DM) 100 mL of distilled water was added (*n* = 2). Samples were homogenized using ultra turrax IKA (14,000 rpm, 2 min IKA-Werke GmbH & Co., KG, Staufen, Germany). The solution was centrifuged at 9000 rpm for 10 min and an aliquot of 25 mL of supernatant was transferred into previously weighted Petri dishes followed by oven-drying at 105 °C for 5 h (Memmert DO6836, Schwabach, Germany). The solubility, expressed in %, was established by calculation of weight difference.

#### 3.3.7. Statistical Analysis

One-way ANOVA was tested for significant differences (*p* < 0.05) among mean values using Statistica 13.1 (StatSoft, Kraków, Poland). Prior to ANOVA, data were first checked for homogeneity of variance, and nonparametric alternatives applied if assumption was violated. Significant ANOVA effects were examined via Tukey’s honestly significant difference (HSD) post hoc test. Pearson correlation coefficients (*r*) evaluated relationships among selected variables, with matrices generated in XLSTAT version 2025.1.1. Principal component analysis (PCA) in XLSTAT further explored dataset patterns and variability.

## 4. Conclusions

This study demonstrates the valorization potential of post-infusion marc obtained from clitoria and borage flowers, establishing a sustainable framework for the recovery and utilization of by-products from herbal extraction. From a technological perspective, powders derived from the marc extract of both flowers exhibited lower water activity and higher solubility, supporting their use in aqueous food systems. In terms of bioactive compounds, approximately half of the phenolic compounds present in the wet marc were recovered in the marc extract fraction, while the remaining portion was retained in the solid residue. The application of more intensive extraction strategies could further enhance phenolic recovery into the solvent, thereby reducing residual phenolic content in the solid fraction. The pre-infusion format also influenced the results, as powders obtained from intact flowers exhibited higher TPC than those derived from milled flowers. Carrier selection played a critical role in powder properties, with by-product powders formulated with inulin showing higher TPC, a greater sum of individual phenolic compounds, enhanced antioxidant capacity, and improved solubility. Clitoria by-products consistently surpassed borage by-products in phenolic content and antioxidant capacity. The clitoria marc exhibited approximately 1.7-fold higher TPC than borage by-products, with antioxidant assays indicating around two-fold higher FRAP and three-fold higher ABTS radical scavenging activity, highlighting pronounced matrix-dependent differences. In conclusion, clitoria post-infusion marc exhibits significant potential for further investigation and transformation into value-added plant-based food ingredients.

## Figures and Tables

**Table 1:** Pearson correlation matrix of key chemical variables in powders derived from clitoria marc extract.

Variables	Sum of Phenolics	TPC	FRAP	ABTS
Sum of phenolics	1	0.788 (*p* = 0.212)	0.714 (*p* = 0.286)	0.840 (*p* = 0.160)
TPC		1	0.929 (*p* = 0.071)	0.984 (*p* = 0.016)
FRAP			1	0.856 (*p* = 0.144)
ABTS				1

**Table 2:** Pearson correlation matrix of key chemical variables in powders derived from clitoria marc residue.

Variables	Sum of Phenolics	TPC	FRAP	ABTS
Sum of phenolics	1	0.868 (*p* = 0.132)	0.967 (*p* = 0.033)	0.947 (*p* = 0.053)
TPC		1	0.827 (*p* = 0.173)	0.949 (*p* = 0.051)
FRAP			1	0.860 (*p* = 0.140)
ABTS				1

**Table 3:** Pearson correlation matrix of key chemical variables in powders derived from borage marc extract.

Variables	Sum of Phenolics	TPC	FRAP	ABTS
Sum of phenolics	1	0.586 (*p* = 0.414)	0.824 (*p* = 0.176)	0.901 (*p* = 0.799)
TPC		1	0.939 (*p* = 0.060)	0.879 (*p* = 0.122)
FRAP			1	0.678 (*p* = 0.322)
ABTS				1

**Table 4:** Pearson correlation matrix of key chemical variables in powders derived from borage marc residue.

Variables	Sum of Phenolics	TPC	FRAP	ABTS
Sum of phenolics	1	0.738 (*p* = 0.262)	0.828 (*p* = 0.171)	0.605 (*p* = 0.395)
TPC		1	0.963 (*p* = 0.037)	0.871 (*p* = 0.129)
FRAP			1	0.929 (*p* = 0.071)
ABTS				1

## Data Availability

Data are contained within the article and Supplementary Materials.

## Supplementary Materials

The following supporting information can be downloaded at: https://www.mdpi.com/article/10.3390/molecules31081335/s1, Figure S1: Representative full-range HPLC chromatogram (0–70 min) of phenolic compounds in the powders derived from intact clitoria marc residue; Figure S2: Expanded HPLC chromatogram (30–60 min) of phenolic compounds in powders derived from intact clitoria marc residue; Table S1: Regression coefficients and statistical parameters of the chlorogenic acid calibration curve; Table S2: ANOVA results for the linear regression model of the chlorogenic acid calibration curve.
