# Supplementary material for: Investigating the Potential of By-Products from Clitoria and Borage Flower Infusions for Valorization: A Comparative Study
Source: Molecules. 2026 Apr 18;31(8):1335. doi: 10.3390/molecules31081335 (PMC13119518; doi:10.3390/molecules31081335)
Supplement: Supplementary file 1 [file molecules-31-01335-s001.zip › molecules-4177970-supplementary.pdf]

A representative full-range HPLC chromatogram (0–70 min) of phenolic compounds identified in powders derived from intact clitoria marc residue is presented in Figure S1. The chromatogram, acquired at 254 nm under gradient elution conditions, illustrates the overall separation profile. Peaks are observed in both the early and middle stages of the run, followed by a gradual baseline shift corresponding to the increasing strength of the mobile phase. An expanded chromatogram covering the retention time window of 30–60 min is shown in Figure S2, providing enhanced resolution of individual phenolic constituents. Multiple well-defined peaks are observed between 38-50 min, indicating the presence of diverse phenolic compounds. A dominant peak is evident at approximately 44–45 min, suggesting the presence of a major phenolic compound.

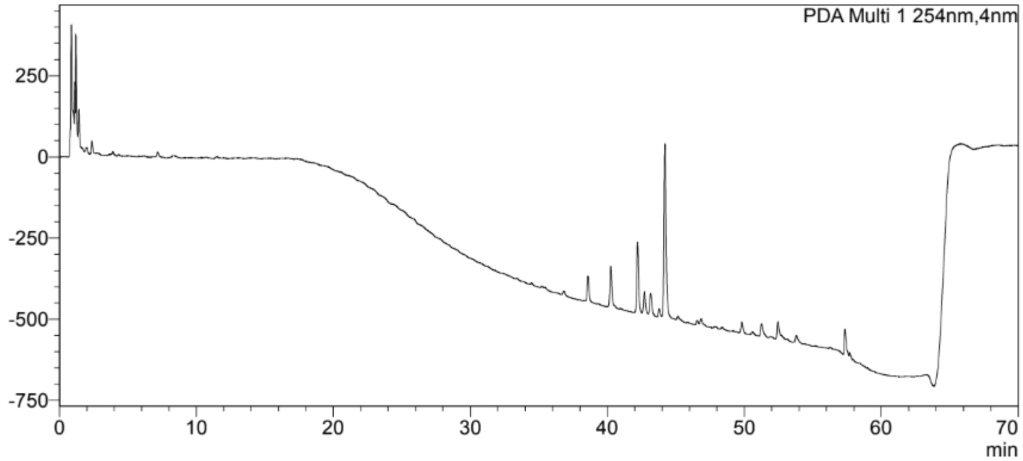

Figure S1. Representative full-range HPLC chromatogram (0–70 min) of phenolic compounds in the powders derived from intact clitoria marc residue

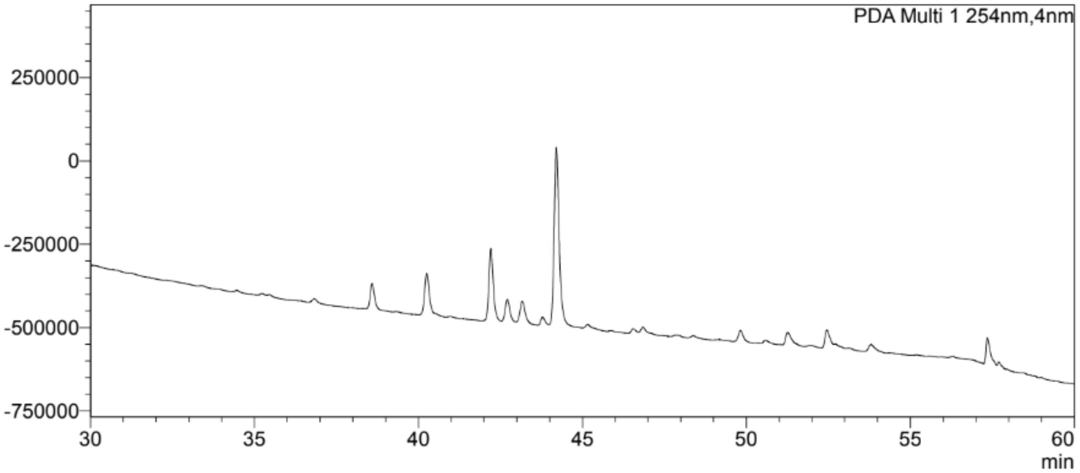

Figure S2. Expanded HPLC chromatogram (30–60 min) of phenolic compounds in powders derived from intact clitoria marc residue

Table S1. Regression coefficients and statistical parameters of the chlorogenic acid calibration curve

Model term	Coefficients	SE	t Stat	P-*value*	Lower 95%	Upper 95%
**Intercept**	-11433.30	13807.73	-0.828	0.418	-40333.20	17466.61
**Slope**	16350523.98	28094.22	581.99	7.437E-42	16291722.10	16409325.86

SE: Standard error

Table S2. ANOVA results for the linear regression model of the chlorogenic acid calibration curve

Source	df	SS	MS	F	Significance F
**Regression**	1	6.30E+14	6.30E+14	338711.04	7.44E-42
**Residual**	19	3,533,524,112	1,859,749,533		
**Total**	20	6.30E+14			

df: degree of freedom, SS: sum of squares, MS: mean squares
